# Microbial Ecology of Sulfur Biogeochemical Cycling at a Mesothermal Hot Spring Atop Northern Himalayas, India

**DOI:** 10.3389/fmicb.2022.848010

**Published:** 2022-04-13

**Authors:** Shekhar Nagar, Chandni Talwar, Mikael Motelica-Heino, Hans-Hermann Richnow, Mallikarjun Shakarad, Rup Lal, Ram Krishan Negi

**Affiliations:** ^1^Fish Molecular Biology Laboratory, Department of Zoology, University of Delhi, New Delhi, India; ^2^UMR 7327, Centre National de la Recherche Scientifique, Institut des Sciences de la Terre D’Orleans (ISTO), Université d’Orleans-Brgm, Orleans, France; ^3^Department of Isotope Biogeochemistry, Helmholtz Centre for Environmental Research – UFZ, Leipzig, Germany; ^4^Evolutionary Biology Laboratory, Department of Zoology, University of Delhi, New Delhi, India; ^5^NASI Senior Scientist Platinum Jubilee Fellow, The Energy and Resources Institute, New Delhi, India

**Keywords:** metagenomics, hot spring, biogeochemical cycle, sulfur spring, evolution

## Abstract

Sulfur related prokaryotes residing in hot spring present good opportunity for exploring the limitless possibilities of integral ecosystem processes. Metagenomic analysis further expands the phylogenetic breadth of these extraordinary sulfur (S) metabolizing microorganisms as well as their complex metabolic networks and syntrophic interactions in environmental biosystems. Through this study, we explored and expanded the microbial genetic repertoire with focus on S cycling genes through metagenomic analysis of S contaminated hot spring, located at the Northern Himalayas. The analysis revealed rich diversity of microbial consortia with established roles in S cycling such as *Pseudomonas*, *Thioalkalivibrio*, *Desulfovibrio*, and *Desulfobulbaceae* (*Proteobacteria*). The major gene families inferred to be abundant across microbial mat, sediment, and water were assigned to *Proteobacteria* as reflected from the reads per kilobase (RPKs) categorized into translation and ribosomal structure and biogenesis. An analysis of sequence similarity showed conserved pattern of both *dsrAB* genes (*n* = 178) retrieved from all metagenomes while other S disproportionation proteins were diverged due to different structural and chemical substrates. The diversity of S oxidizing bacteria (SOB) and sulfate reducing bacteria (SRB) with conserved (r)*dsrAB* suggests for it to be an important adaptation for microbial fitness at this site. Here, (i) the oxidative and reductive *dsr* evolutionary time–scale phylogeny proved that the earliest (but not the first) *dsrAB* proteins belong to anaerobic *Thiobacillus* with other (*rdsr*) oxidizers, also we confirm that (ii) SRBs belongs to δ-*Proteobacteria* occurring independent lateral gene transfer (LGT) of *dsr* genes to different and few novel lineages. Further, the structural prediction of unassigned DsrAB proteins confirmed their relatedness with species of *Desulfovibrio* (TM score = 0.86, 0.98, 0.96) and *Archaeoglobus fulgidus* (TM score = 0.97, 0.98). We proposed that the genetic repertoire might provide the basis of studying time–scale evolution and horizontal gene transfer of these genes in biogeochemical S cycling.

## Introduction

The untapped sulfur (S) compounds oxidizing microorganisms (SOM) and S compounds reducing microorganisms (SRM) microbial communities residing in extreme and contaminated environmental conditions such as hot water, sulfide contaminated springs offer an intriguing opportunity to explore the unique microbial diversity with uncovered metabolic potential (Ghilamicael et al., 2017). The investigations of such microbiota began with focus on identifying and culturing novel thermostable biocatalysts with huge biotechnological applications (Inskeep et al., 2010; Li et al., 2014; Ayangbenro and Babalola, 2017). However, little progress has been made in exploring the correlation between microbiome and geochemistry of hot spring systems particularly that possess mesothermic hot waters with neutral pH and elemental S and sulfate richness (Ghosh et al., 2012; Roy et al., 2020). Moreover, the survival of microbes in these niches is often supported by community dynamics and interactions. Studies of such ecosystems may provide insights into the microbial evolution of specific pathways for microbial biogeochemical cycling of minerals. However, with about 400 thermal hot water springs located in India, less than 15% have been explored for biogeochemical and taxonomical classification using genomics and metagenomics approaches (Cinti et al., 2009; Saxena et al., 2017). Sulfur springs provide harsh physiochemical conditions to sustain the growth of only meso- and hyper-thermophilic microbes which includes S oxidizers and sulfate reducers (Chan et al., 2015; Gonsior et al., 2018). The survival could also be achieved with “microorganism adaptation” by several resistance mechanism such as activity of bioprecipitation, biosorption, extracellular sequestration, and/or chelation (Haferburg and Kothe, 2007). During these changes, the exchange of genetic material by means of horizontal gene transfer (HGT) is prevalent and necessary for the adaptation of microbes through the acquisition of novel genes.

Khirganga, the mesothermal S spring in Northern Himalayas discharging waters rich in sulfate, chlorine, sodium, and magnesium ions has remained uncharted so far (Shirkot and Verma, 2015; Poddar and Das, 2018). High levels of sulfides in the environment accounts for the milky appearance of the hot spring water with white microbial mats predicted to be formed from sulfide reduction by the S-related prokaryotes (SRP) enriched at this site (Sharma et al., 2004; Dong et al., 2019). The microbial S disproportionation one of the oldest (about 3.5 billion years ago; Finster, 2011) biological processes on Earth producing sulfide, sulfite, and sulfate compounds establishes a complex network of pathways in the biogeochemical S cycle. Thus far, it is the very foremost metagenomic investigation of microbial communities in Khirganga (average atmospheric temperature 6.9 ± 0.3) focused on exploring the microbial biogeochemical S cycling with a complex of disproportionation of elemental S conforming intermediary compounds. The current study was carried out *via* microbial mats, sediments, and hot spring water samples in hot spring to decipher the stabilized and diversified genes involved in S cycle intermediary process in anoxygenic, photolithotrophic and chemolithotrophic S-oxidizing and reducing bacteria (Dahl and Truper, 1994; Hipp et al., 1997). The work expands the genetic and evolutionary information for S cycling genes and evaluates the biodiversity and applications for screening of the novel thermostable enzymes from microorganisms. Further, understanding these adaptations *vis-à-vis* the physiological properties and metabolic processes in these springs could be monitored as the engineered SRP consortia could develop into an effective tool in optimizing degradation of sewage waste in industrial processes (Ayangbenro et al., 2018). Also, the sulfate reducing bacteria (SRB) implied to treat various environment contaminants including metals (Mothe et al., 2016; Zhang et al., 2016), metalloids (Battaglia-Brunet et al., 2012; Sahinkaya et al., 2015), various non-methane hydrocarbons (Callaghan et al., 2012), alicyclic hydrocarbons (Jaekel et al., 2015), nitroaromatic compounds (Boopathy, 2014; Mulla et al., 2014), and aromatic hydrocarbons (Stasik et al., 2015; Meckenstock et al., 2016; Kamarisima et al., 2019).

## Materials and Methods

### Sample Collection, Physicochemical Analysis, and Helium Ion Microscopy

Samples of microbial mat deposits (250 g), sediment (250 g) and water (5L) were collected from Khirganga hot water spring (31°59′34″ N, 77°30′35″ E) in February 2017. Sampling was performed in two replicates for each habitat from two closely located primary thermal outlets (31°99′18″ N, 77°50′96″ E) and secondary outlets (31°99′19″ N, 77°50′96″ E). The surface temperature and pH of each habitat were recorded on site.

First, microbial mats and sediment were digested in pure nitric acid and water samples were filtrated to 0.1 μm prior to chemical analysis. All samples were subjected to physicochemical analysis for major elements. Concentrations of major cations (Na^+^, K^+^, Mg^2+^, and Ca^2+^) and anions (SO_4_^2–^ and Cl^–^) were analyzed by ionic chromatography (Dionex ICS-2000, Sunnyvale, CA) using the columns CS16A for measuring cations and AS17 for anions. An elemental analysis of minor and trace elements through inductively coupled plasma mass spectrometry (ICP-MS) Agilent ICP-MS 7,900 with ultra-high matrix introduction (UHMI). The samples of sediments and microbial mats were desiccated overnight followed by ethanolic dehydration and microstructure was studied using a scanning electron microscope at the Center for Chemical Microscopy (ProVIS). Images were captured using a high efficiency detector.

### Metagenomic DNA Extraction, Sequencing, and Assembly

For the extraction of total DNA from microbial mats, 0.25-g samples were processed following a method described by Varin et al. (2010). The total community DNA from 0.25-g sediment samples and 5 L of filtered water (0.45 μm) were extracted using PowerMax Soil DNA isolation kit (MoBio Laboratories Inc., Carlsbad, CA, United States) following the manufacturer’s instructions. Sequencing was performed at Beijing Genome Institute (BGI), Hongkong, China using Illumina Hiseq 2,500 platform. Paired end libraries of read length 100 base pairs (bp) were generated with insert size of 350 bp. The raw sequences were quality filtered using SolexaQA (Cox et al., 2010), and the low-quality sequences below *Q*_20_ quality cut-off and artificially duplicated reads (ARDs) were castoff using Illumina-Utils (Eren et al., 2013) and duplicate read inferred sequencing error estimation (DRISEE) (Gomez-Alvarez et al., 2009), respectively. Further, the assembly was integrated in IDBA-UD (Peng et al., 2012) with 50-bp insertion length, minimum *k-mer*: 31, maximum *k-mer*: 93 (61 for water) using seed *k-mer* size for alignment 30 bp and the minimum size of contig as 200 bp while allowing minimum multiplicity for filtering *k-mer* while building the graph.

### Taxonomic and Functional Assignments

Alpha diversity within each sample was estimated as abundance-weighted average of annotated species from source databases built in MG-RAST v3.0 (Meyer et al., 2008) and expressed as Shannon diversity index transformed based on rarefaction curve using the following formula:


log(α-diversity)10/ln(10)

Diversity at phylum level was inferred from MG-RAST (maximum *e*-value, 1 × 10^5^ and minimum percentage identity cutoff, 60%). A paired-sample *t*-test was applied on the phylum determined in any habitat pair to estimate significant similarities based on taxonomic mean abundance using SPSS (SPSS Inc., version 20.0, IBM). Microbial genera were deciphered based on clade-specific markers to identify taxonomy up to species level using MetaPhlAn v2.0 (Truong et al., 2015) and a heatmap was constructed using Bray–Curtis dissimilarity with supporting dendrograms for both species and samples. We used HUMAnN2 (Franzosa et al., 2018) to perform phylum-resolved functional profiling of the communities that maps contigs onto the pangenomes of the known species of the community and quantifies the pathways and UniRef90 gene families database (Suzek et al., 2015). Later, these UniRef90 families were regrouped as clusters of orthologous groups (COGs) annotations based on eggNOG (Huerta-Cepas et al., 2017). Open reading frames (ORFs) of assembled metagenome were predicted using Prodigal v2.6.1 (Hyatt et al., 2010) and annotated at hierarchy levels, namely, subsystems, protein families, and individual enzymes using Prokka v1.12 (Seemann, 2014). The amino acid sequences were mapped against Kyoto Encyclopedia of Genes and Genomes (KEGG) database (Kanehisa et al., 2004) and top 50 metabolic pathways in all of the six samples were compared through heatmap constructed using package *pheatmap* (Kolde and Kolde, 2015) and *ggplot2* (Wickham, 2009) in R (R Development Core Team, 2011). Identification of S substrates disproportionation genes was performed by mapping all predicted ORFs on the HMM databases obtained from TIGRfam v10 (Haft et al., 2012) and Pfam (Finn et al., 2014) using hmmscan v3.1b2 (Eddy, 2011). An abundance of each enzyme was plotted as number of copies annotated within each sample. The sequences with more than 150 amino acids were queried against the National Center for Biotechnology Information (NCBI) Microbial proteins from RefSeq *nr* database (04 April 2020) using BLASTp (Altschul et al., 1990) to identify the sequences producing significant alignments for taxonomic confirmation.

### Analysis of Diversity of Sulfate Reduction Proteins

For sequence similarity networks (SSN), amino acid sequences of sulfide oxidation and sulfate reduction proteins annotated in all six samples annotated by KEGG Ids were implied over an empirical measurement of diversity. For this, an all-vs.-all BLAST was performed to define the similarities/variations between sequence pairs of diversifying sulfate reduction proteins. A user defined threshold was optimized according to the alignment score and maximum length of BLAST results in diversifying and stabilized protein sequences. Clustering was performed using CD-HIT (Li and Godzik, 2006) on the scores of BLASTp pairwise alignments at a threshold value (*e*-value of 1*e*-30). The networks were visualized in Cytoscape v3.7.1 (Shannon et al., 2003). The average number of degree and neighbors for a protein sequence or a node was calculated as:


$$k=\frac{2k}{N}$$

where, *K* is denoted with number of edges and *N* is denoted with total number of nodes. Also, to determine the divergence/similarity among nodes or protein sequences was calculated as:


$$D=\frac{2k}{N\left(N-1\right)}$$

The attributes of node degree distribution, average clustering coefficient, average neighborhood connectivity, and closeness centrality were studied through power law fits to determine their correlation with number of neighbors. Sulfur oxidizing bacteria (SOB) and SRB were identified for the sequences that could be classified up to genus level to study the distribution of S substrates oxidation and reduction genes in the different clusters.

### Sequence Alignment, Phylogeny and Structure Prediction of Putative Unidentified Dsr and Asr Enzymes

To elucidate the phylogeny of key sulfite reductases, the DsrA/B and AsrA/B protein sequences (more than 150 amino acids) were individually aligned using MUSCLE v3.8.31 (Edgar, 2004) and clustered using UPGMB (unweighted pair group method with arithmetic mean). All the alignments were end trimmed manually and maximum likelihood (ML) phylogeny was inferred with 500 bootstrap resampling using RAxML v8.0.26 (Stamatakis, 2014). For this, we used standalone version of RAxML which was called as follows:


raxmlHPC-PTHREADS-sinput-N 500-nresult-fa-p 12345-x 12345-mPROTGAMMAGTR.

The resulting phylogenies were also confirmed using most complex general time-reversible model (CAT-GTR; Tavaré, 1986) with PhyloBayes v1.7b using CIPRES Science Gateway v 3.3 (Lartillot et al., 2009) that incorporates different rates for every change and different nucleotide frequencies.

For proteins showing similarity with those from uncultured bacteria, we determined the structures using I-TASSER suite (Yang and Zhang, 2015). These predicted structures were then aligned onto their top structural analogs and C-scores, TM-scores, and RMSD were computed and ligand binding sites with conserved residues were identified. The TM-score is to compare two models based on their given residue equivalency (i.e., based on the residue index in the PDB file). It is usually NOT applied to compare two proteins of different sequences. The TM-score predicted from structural alignment of two proteins while comparing them based on residue equivalency such that a score of 0.6 and above denote the two proteins to be fairly aligned (Yang and Zhang, 2015). The TM-align will first find the best equivalent residues of two proteins based on the structure similarity and then output a TM-score.

## Results

### Description of Sampling Site and Microscopic Analysis of Samples

**FIGURE 1:**
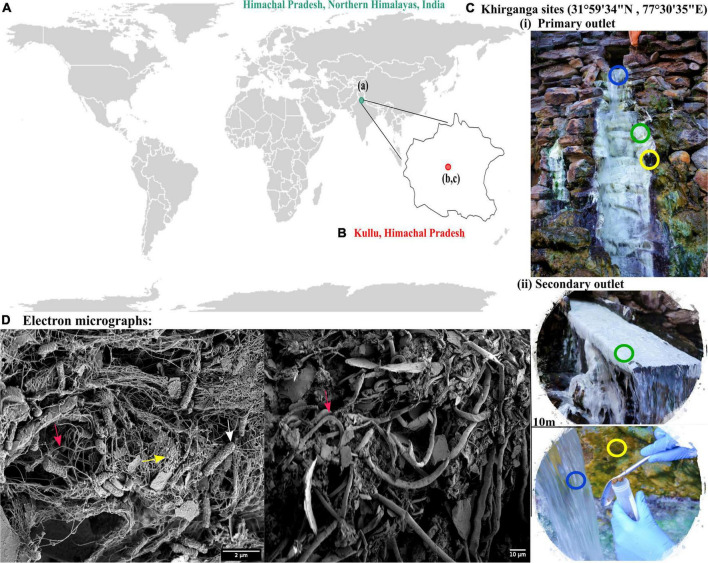
**(A)** Geographical location of Himachal Pradesh, Northern Himalayas. **(B)** Khirganga hot spring as shown in the maps are located in Parvati valley in Kullu district of Himachal Pradesh, India. **(C)** The different habitats from where the samples were collected are shown: microbial mat (green), sediment (yellow), and water (blue). Samples were collected in replicates from two outlets located 10-m distance apart. **(D)** The scanning electron micrographs of different habitat samples shown with arrow demarcating the filamentous (cyanobacteria), cocci-shaped and rod-shaped bacteria (SRB) in pink, yellow, and white, respectively.

The Khirganga is a natural hot spring setting that lies in the Parvati Valley in the Northern hemisphere of the great Himalayas (31°59′34″ N, 77°30′35″ E, altitude 2,978 m) at district Kullu, Himachal Pradesh, India (Figures 1A,B). For this study, samples from all three habitats, namely, microbial mats, sediments, and water were collected proximal to the major opening (KgM1, KgS1, KgW1) and from a distance of 10 m (KgM2, KgS2, KgW2) as shown in Figure 1C.

Using an electron microscopy, we dissected the microstructure of the niches and were able to visualize cellular structures on complex sample matrices. The microbial diversity was visualized as numerous filamentous structures in microbial mats and sediments that resembled Cyanobacteria. In addition, rod and cocci shaped cells of varying sizes were also observed in the sample matrices that providing a visual insight into the microbial diversity at this mesothermic site (Figure 1D).

### Physicochemical and Elemental Analysis

The *in-situ* measures of water temperature were from 59°C at the outlet to 55°C at 10 m distance (Table 1). Microbial mat deposits and sediments had much lower temperature (42–45°C) than water. The pH of the hot spring water was 6.7 while sediments and mats were slightly acidic with pH 6.1 and 6.3, respectively. Thus, all three habitats were recorded to be mesothermic. The physicochemical composition of the hot spring is dominated by anions of chloride (up to 11,024 ug/g) and sulfate (up to 10,079 ug/g) while ions of calcium and potassium were abundant (Table 1 and Supplementary File 1). Importantly, sulfates (SO_4_^2–^) concentration in microbial mats and sediments were higher (9,529 ± 313.29 ug/g; 10,079 ± 863.29 ug/g, respectively) and exceeded the limit of 8,000 ug/g standardized by Environment Protection Act (EPA, 2001) and also found to be exceeded the limit of 53 mg/L in surface waters (79.82 ± 1.85 mg/L) (EPA, 2001; Table 1). The chlorides (1,456.77 ± 367.27 mg/L), manganese, sodium, and silicon constituents in the hot spring waters were surpassing the normal average concentrations of 250, 0.05, 200, and 4 mg/L, respectively (EPA, 2001) in surface water samples (Supplementary File 1). Among others, the predominant elements and minerals in water samples were aluminum, magnesium, copper, zinc, and arsenic.

**TABLE 1:** Physicochemical and elemental analysis of the niche samples.

Value for indicated sampling sites			
Environmental data	Mat	Sediment	Water
Temperature (°C)	42.4 ± 0.56	44.55 ± 1.06	57 ± 2.2
pH	6.2 ± 0.14	6.5 ± 0.14	6.7 ± 0

**Major ions/metals**	**(μg/g)**	**(μg/g)**	**(mg/L)**

Cl^–^	11024.88 ± 983.17	15547.08 ± 204.96	1456.77 ± 367.27
SO4^2–^	9529.06 ± 313.29	10079 ± 863.29	79.82 ± 1.85
Na^+^	976.88 ± 64.83	991.47 ± 7.88	1338.52 ± 167.97
Ca^2+^	7529.71 ± 1034.68	5111.4 ± 588.21	30.27 ± 7.32
K^+^	3681.66 ± 886.65	7743.91 ± 412.12	41.56 ± 9.03
Al	7205.41 ± 1685.37	22011.1 ± 2312.9	0.05 ± 0
Mg	3528.62 ± 735.04	8204.28 ± 964.02	8.83 ± 0.02
Si	3.22 ± 0.66	5.28 ± 0.42	31.5 ± 1.36
Mn	1149.7 ± 178.85	1219.29 ± 13.93	0.34 ± 0.03
Cu	14.4 ± 2.62	27.23 ± 1.71	<0.01
Zn	27.44 ± 6.36	67.35 ± 0.47	<0.01
As	3.9 ± 0.64	6.87 ± 0.22	<0.01
Ag	5.51 ± 3.7	5.1 ± 0.03	<0.01
Pb	17.74 ± 2.54	11.4 ± 1.27	<0.00

*Values for microbial mats and sediments samples are given in (μg/g) and those of water samples are shown in parts per million (ppm).*

### Metagenomic DNA Sequencing and Assembly

A large metagenomic dataset was obtained from sequencing having number of reads sized up to ∼18 Gb for each sample. We retrieved a total number of reads ranging between 1.1 × 10^8^ to 1.5 × 10^8^ in all samples which were assembled into 180,849-519,194 (more than 200 bp) contigs. After assembly, the metagenomes sizes varied between 329 and 600 Mbp. A summary of characteristics of the datasets and assembled metagenomes is provided in Table 2. The alpha diversity estimated as the Shannon diversity indices ranged between 2.5 and 3 (Table 2 and Supplementary File 2).

**TABLE 2:** Characteristics of sequenced datasets generated and assembled metagenomes obtained for each sample from different habitats.

Attributes	Microbial mat		Sediment		Water	
Sample name	KgM1	KgM2	KgS1	KgS2	KgW1	KgW2
(Clean reads × 100) bases	110,861,650 × 100	152,895,302 × 100	148,273,248 × 100	145,473,498 × 100	144,915,928 × 100	141,139,772 × 100
No. of contigs	180,849	216,350	404,684	398,499	519,194	506,942
Size of assembly (Mbp)	329	397	592	583	600	591
*N*_50_	7,668	8,123	2,672	2,664	3,945	3,985
GC-content (%)	55.55 ± 12.97	54.0 ± 12	59.70 ± 10.74	59.61 ± 11	56.43 ± 12.97	56.34 ± 13
Shannon diversity index	2.6	2.6	2.7	2.7	3.0	3.03
Paired-sample *t*-test (*p*-value < 0.05)	0.194		0.334		0.416	
Predicted proteins	305,932	369,390	570,660	562,428	545,606	537,498
CRISPRs	452	595	560	570	1,105	1,081

### Microbial Consortia and Proportionality of S Oxidizing Bacteria and Sulfate Reducing Bacteria

**FIGURE 2:**
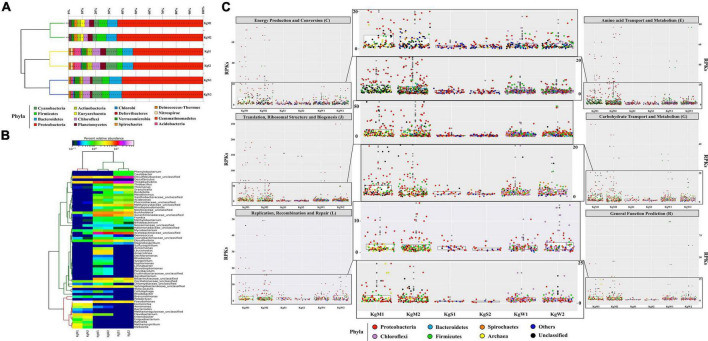
Relative abundance of phylum and genera. **(A)** The stacked bar representation shows the dominating phylum in all three habitats. The dendrograms show hierarchical clustering between species and samples. **(B)** The abundant common genera *Pseudomonas* (20–60%), *Desulfobulbaceae_unclasssifed* (15–20%), *Burkholderia*, *Desulfovibrio*, and *Thioalkalivibrio* (1–20%) are shown here relative abundance × log*_*x*_* scale. **(C)** Taxa based functional profiles demonstrating the major phylum contributing toward COG subsystems and percentage of proteins annotated within each COG category for the habitat sites. The distribution of the RPKs were mapped in accordance to the member abundance in the habitats. Dots were colored according to the phylum.

Bacteria belonging to 15 different phyla dominated the microbial communities. The average percentage relative abundances of major phyla in the three habitats shown in parentheses in the order microbial mat, sediment, and water is as follows: *Proteobacteria* (62.1, 50.5, and 58.7%), *Bacteroidetes*, *Firmicutes*, *Cyanobacteria, Planctomycetes*, and *Chloroflexi* (Figure 2A). Species belonging to phylum *Proteobacteria* are found in varied temperature ranges which results in their dominance in various hot springs (Bowen de León et al., 2013; Singh and Subudhi, 2016; Saxena et al., 2017) and disproportionation of S compounds is mainly carried out by SRM of *Proteobacteria* (Finster, 2011), Besides, *Actinobacteria*, *Spirochaetes*, *Verrucomicrobia, Acidobacteria, Deinococcus-Thermus, Deferribacteres, Chlorobi, Gemmatimonadetes*, and *Nitrospirae* were also detected in all three habitats with relative abundances less than 3%. Among the three habitats, microbial diversity profiles of water and sediments were more similar compared to those of microbial mats.

The highest genus level diversity was revealed in sediment (*n* = 196) followed by water (*n* = 132) and mat (*n* = 63). The top-50 genera in all habitats were plotted (Figure 2B). The microbial mats were dominated by *Pseudomonas* (51.8%) followed by unclassified genera of family *Desulfobulbaceae* (SRB; 5.2%) and *Flavobacterium* (4.3%). Among the abundant genera in the microbial mats were *Thioalkalivibrio* (1.3%, SOB), *Aeromonas* (1.3 %), *Klebsiella* (1%), *Exiguobacterium*, *Enterobacter*, and *Escherichia* (more than 1%) (Figure 2B). On the other hand, sediment habitats were found to be enriched in *Thioalkalivibrio* (SOB; 18.9%), *Desulfobulbaceae* (SRB; 9.9%), *Halothiobacillus* (8.3%), *Burkholderia* (7.1%), and unclassifed genera of families *Acetobacteraceae* (6.1%). The hot spring waters with highest diversity of bacterial genera were dominated by *Thioalkalivibrio* (SOB; 20.5%), *Acetobacteracea* (9.6%), and *Desulfovibrio* (SRB; 5.9%). Other genera with less than 6% abundances in all three habitats were also detected as shown in Figure 2B.

### Metabolic Functions of the Community and S Disproportionation Genes

The major gene families inferred to be abundant across all three habitats were assigned to *Proteobacteria* followed by *Chloroflexi*, *Firmicutes*, *Bacteroidetes*, and *Spirochaetes* as reflected from the reads per kilobase (RPKs) in the metagenomes. These gene families were then regrouped as COGs and the top functions were determined to be translation, ribosomal structure and biogenesis (COG: J), amino acid transport and metabolism (E), general function prediction (R), energy production and conversion (C), replication, recombination and repair (L), and carbohydrate transport and metabolism (G). These functions in microbial mats were carried out by *Proteobacteria* (COGs: J, E, C, and G) and unclassified bacteria (COGs: R and L); in sediments by unclassified bacteria (COG: J) and *Proteobacteria* (COGs: E, R, C, L, and G) and in water by *Proteobacteria* (COGs: J and C), *Firmicutes*, and *Chloroflexi* (COGs: E, R, G, and L) (Figure 2C and Supplementary File 3).

**FIGURE 3:**
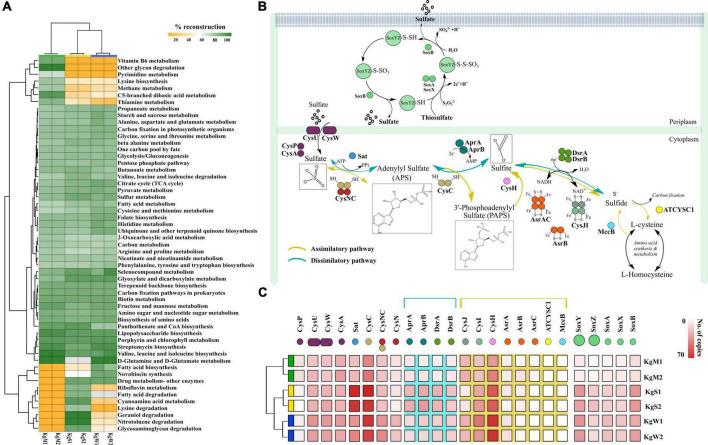
**(A)** Reconstruction of top 50 pathways annotated using KEGG automatic annotation server. Heatmap matrix representation and clustering was performed by using “pheatmap” package (Kolde and Kolde, 2015) in R (R Development Core Team, 2011). **(B)** The sulfate reduction pathway involved a group of reductases, kinases, and transferases with the product chemical structures generated through chemDraw7 and Inkscape v0.9 (Inkscape Project, 2020). **(C)** The gene copy number of both sulfate reduction and sulfide oxidation pathway that were partitioned in different habitats showed here using ggplot2 in R (R Development Core Team, 2011).

The ORFs that were categorized on the basis of KEGG categories were mapped onto the metabolic functions and the pathways that could be reconstructed with more than 60% completeness were used to define the metabolic potential of the habitats. Based on this criterion, we studied the top-50 functional pathways of each habitat and identified the core functions (*n* = 37 pathways) of the communities that included the common pathways for metabolism of nucleotides, carbohydrates, and amino acids (Figure 3A and Supplementary File 4). In addition, we determined differentially abundant pathways in each habitat: microbial mats (*n* = 7), sediments (*n* = 7), and water (*n* = 2). The community functional profiles of sediment and water were more similar compared to those of microbial mats which may be due to the stratified layered organization of the mats which are different in sediment and water. The microbial communities in mats were optimized for metabolism of methane specifically, members of genus *Methanospirillum*, which were abundant in mats (Figure 2B). Other metabolic pathways such as lysine biosynthesis, C5-branched dibasic acid metabolism, thiamine metabolism, pyrimidine metabolism, vitamin B6 metabolism, and other glycan degradation were also found to be abundant in microbial mat.

The S metabolism pathway could be reconstructed within a range of 76.31–84.21% which was maximum in sediment and minimum in microbial mat. A total of 75 genes were responsible for S metabolism present in all the samples with a mean copy number of 1580 ± 249.2. To gain insights into S disproportionation potential across all the habitats, we mapped the TIGRfam and Pfam (Supplementary File 4) ids of the 25 associated genes (mean copy number of 980 ± 222.1) on to the ORFs and copy numbers of these genes involved in S oxidation, sulfide oxidation, and sulfate reduction (as described in Figure 3B) were estimated. Thiosulfate ions are fused to the carrier complex of S-oxidizing proteins *soxYZ* (*soxY* = 145, *soxZ* = 71), while L-cysteine S-thiosulfotransferase (*soxX* = 76, *soxA* = 86) and S-sulfosulfanyl-L-cysteine sulfohydrolase (*soxB* = 145) mediate the hydrolytic release of reduced S ions from S-bound *soxYZ*. Besides, the dissimilatory sulfite reductase (DsrAB) encoding genes: *dsrA* = 95, *dsrB* = 83 and anaerobic sulfite reductase subunits *asrA* = 44, *asrB* = 30, *asrC* = 9 reduces sulfite to sulfide (Supplementary File 4). The other 15 genes for reduction of sulfate ions included solute binding protein (*cysP* = 37), ATP binding protein (*cysA* = 168), transport system permease proteins (*cysU* = 158, *cysW* = 154), ATP sulfurylase (*sat* = 254), transferases (*cysC* = 348, bifunctional *cysNC* = 155, *cysN* = 75), phosphoadenosine phosphosulfate reductase (*cysH* = 314), adenylylsulfate reductase subunit A and B (*aprA* = 88, *aprB* = 104), sulfite reductase flavoprotein alpha-component (*cysJ* = 111), sulfite reductase hemoprotein beta-component (*cysI* = 180), homocysteine desulfhydrase (*mccB* = 6), and cysteine synthase (*ATCYSC1* = 4). Figure 3C shows the comparative abundances of these proteins in the three habitats. Here, the results revealed that the microbial S disproportionation occurs largely through the dissimilatory pathway carried out by DsrAB as compared to assimilatory sulfite reductase (AsrABC) mediated reduction. In nature, the dissimilatory pathway is shorter and thus, preferred route of microbial sulfate reduction (Figure 3B; Kushkevych et al., 2020).

**FIGURE 4:**
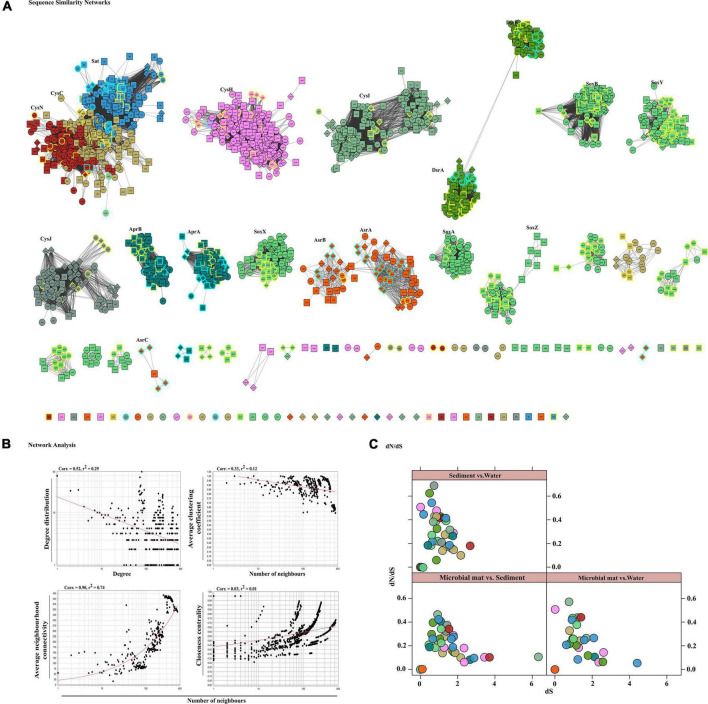
Sequence similarity network analyses. **(A)** Diversity of sulfate reduction genes of both assimilatory and dissimilatory pathways in microbial mat (diamond-shaped), sediment (square), and water (spherical) habitats visualized in cytoscape v3.7.1. Highlighted only the classified taxa, where color cyan belong to SRBs and yellow to SOBs. The network was set at threshold *e*-value cutoff of 1*e*-30 and nodes represent sequences connected through edges if the similarity exceeds the cutoff score. Here, the clusters and isolated nodes were showing the conserved pattern and diversified pattern of the proteins significantly playing an important role in sulfate reduction. **(B)** Topological properties of the similarity networks: degree distribution, average clustering coefficient, average neighborhood connectivity, and closeness centrality are plotted against the number of neighbors. The power law fit curves are shown within each graph. **(C)** Habitat vs. habitat *dN/dS* values of all S cycle genes were estimated and plotted using *xy*-plot in R (R Development Core Team, 2011).

We identified the sequences of S disproportionation producing significant alignments from the *nr* database for taxonomic confirmation and assigned each sequence that could be classified up to genus level to either SOB or SRB (Figure 4A and Supplementary File 5). The taxonomy and evolutionary phylogenetic topologies are discussed in detail in the next section.

### Diversification and Evolution of S Disproportionation Proteins

To gain insights in the differentiation of S disproportionation genes, we study the diversity and evolution of the key enzymes of SOB and SRB communities in this environmental biosystem. Therefore, we employed a two-step strategy of comparing similarities of all sequences in a pairwise fashion through SSN analysis and further estimated the measures of the rates of non-synonymous to synonymous substitutions in their orthologous proteins between each pair of habitats. SSN effectively resolves the pairwise similarities of each sequence (node) with every other sequence of an enzyme or a group of enzymes for a pathway such that any two nodes are connected by edges only if they share sequence homology above a certain cutoff (here *e*-value of 1*e*-30). Thus, SSN provides for an accurate placement of a sequence among its putative homologs (Talwar et al., 2020).

Here, we examined the diversity among 19 key S substrates oxidizing and reducing proteins determined from the communities as shown in Figure 3C, except membrane permeases (CysPUWA) and genes for cysteine synthesis (MccB, ATCYSC1). In total, we retrieved 2,413 protein sequences (mean; *M* = 254, *S* = 480, *W* = 472) denoted by nodes in SSN. The network was organized into 88 connected components including 46 isolated nodes with an average clustering coefficient of 0.84. The connected components were represented by the homologous and heterologous clusters depending on whether they were constituted by the same gene or a number of different genes involved in a pathway, respectively. The number of connected components formed through SSN analysis of S metabolic proteins distributed into gene clusters and the isolated nodes denoted the diverging and highly diverged sequences, respectively (Figure 4A). Hence, we looked into these components to study the diversity of each gene that were distributed as shown in Table 3. The proteins CysNC, CysH, CysI, and CysJ catalyze important steps and act as cofactors for the AsrABC which were all found to be diverging with many isolated nodes and loosely connected components. The enzymes for S oxidation (Sox) were also found to be diverging as observed from loosely formed clusters. On the other hand, all sequences of the key enzyme of dissimilatory pathway, DsrAB, formed only one connected component, which suggested that they might be under convergent evolution at this site (Figure 4A). Further, we compared the distribution of diverged sequences that could be separated as isolated nodes and found that hot spring sediments harbored a high diversity of these enzymes (*n* = 23) in comparison with microbial mats (*n* = 12) and water (*n* = 11).

**TABLE 3:** Attributes of the SS N and *dN*/*dS* analysis of S metabolism genes.

Network parameters	Network analysis of homologous and heterologous clusters															
Sulfate reduction genes		CysNC	Sat	CysH	DsrAB	CysI	CysJ	AprA	AprB	AsrAB	AsrC	SoxA	SoxB	SoxX	SoxY	SoxZ
No. of nodes	2,413	578	254	346	178	170	102	87	98	68	9	86	145	76	145	71
No. of edges	1,62,389	–	–	–	–	–	–	–	–	–	–	–	–	–	–	–
Average degree	134.59	264.2	205.1	121.2	77.8	65.2	57.1	72.4	61.0	17.8	3.4	35.3	119.6	52.1	43.3	21.7
Connected components	88	17		14	1	11	8	2	3	6	4	4	2	2	7	7
Isolated nodes	46	12		8	0	10	5	1	0	4	2	0	0	0	2	2
Network density	0.05	–	–	–	–	–	–	–	–	–	–	–	–	–	–	–
Characteristic path length	1.89	–	–	–	–	–	–	–	–	–	–	–	–	–	–	–
Shortest path	14%	–	–	–	–	–	–	–	–	–	–	–	–	–	–	–
Network centralization	0.16	–	–	–	—-	–	–	–	–	–	–	–	–	–	–	–
Clustering coefficient	0.84	0.88	0.86	0.80	0.94	0.85	0.86	0.93	0.88	0.79	0.76	0.97	0.91	0.89	0.83	0.83
Core genes clusters	–	14	6	9	5	3	2	1	2	1	0	2	4	3	6	2
Range *dN/dS* (≤0)	–	≥0.4	≥ 0.5	≥0.5	≥0.6	≥0.5	≥0.6	≥0.4	≥ 0.1	0	–	0	≥0.4	≥0.1	≥0.3	≥ 0.3

The node degree distribution estimated to be decreasing with increasing protein quantity (correlation = 0.52, *r*^2^ = 0.29), average neighborhood connectivity within the networks interpreted as function in *k* was increasing and positively correlated (correlation = 0.90, *r*^2^ = 0.74) (Figure 4B). Furthermore, closeness centrality curve that measures closeness between nodes was unable to reach the bench top (correlation = 0.03, *r*^2^ = 0.01), might be due to the maximum number of connected components and less sequence homology. So, we also analyzed each protein cluster individually by using network analysis, 178/2,413 nodes of the network DsrAB protein cluster found to be conserved showed higher clustering coefficient values (0.94), followed by AprA (0.93), AprB, CysNC (0.88), and others (Table 3). The evolutionary selection pressures on these genes were studied through estimation of *dN*/*dS* values calculated for a subset of conserved gene sequences in all three habitats (Figure 4C). The number of core genes and the range of *dN*/*dS* values identified for each gene are shown in Table 3. The *cysJ* and *cysI* genes were found to be under moderate selection pressures with *dN*/*dS* values in the range 0.4–0.7 (Figure 4C and Supplementary File 6). The results supported the observation as these enzymes code for important co-factors for the AsrABC that were found to be diverging in through SSN analysis. Therefore, the microbial genes for assimilatory reduction pathway are diversifying under moderate selection pressures.

### Divergence, Phylogeny, and Structural Relationships of Dissimilatory Sulfite Reductase and Assimilatory Sulfite Reductase

The enzymes catalyzing the reductive (Dsr) or oxidative (rDsr) transformation between sulfite and sulfide appear to be related with respect to their subunit composition and catalytic properties (Loy et al., 2009). The *dsr* genes have been characterized from bacterial as well as archaeal domains (Chang et al., 2001; Grim et al., 2011; Colman et al., 2020). However, their evolution in these domains has long remained a subject of discussion. Our preliminary results showed that both subunits of *dsr* genes (*dsrA* = 79, *dsrB* = 72) corresponds to about 70 newly identified organism for both oxidation and reduction processes (Supplementary File 8). Through RAxML phylogenetic analysis, it can be confirmed that the *dsrAB* genes have been introduced in most of the newly identified members by a multiple independent LGT (Anantharaman et al., 2018). Importantly, organisms from *Acidobacteria*, *Candidate division Zixibacteria*, *Chloroflexi*, and β-*Proteobacteria* form completely novel lineages other than known DsrAB clusters identified through RefSeq *nr* protein database with accession numbers (National Center for Biotechnology Information; Supplementary File 5). Hence, the dsr from sulfate reducers formed a separate cluster, with sequences from *Desulfarculus, Desulfocarbo, Desulfarcinum, Thermodesulfobacteria, Syntrophobacter, Desulfomonile, Desulfovibrio, Desulfatirhabdium*, and *Desulfobacteriaceae* in both DsrA and DsrB phylogenies and additionally, *Dissulfuribacter* in DsrB (Supplementary File 8). We proposed that these organisms with newly identified lineages of *dsr* genes involved in sulfite/sulfate oxidation and reduction likely serves an important control on S cycling on terrestrial subsurface. The divergence of *dsrAB* between unrelated taxa could be driven through combination of speciation, functional diversification, and LGT. Also, there is equal possibility of non-functionality of the genes in these taxa (Loy et al., 2008; Anantharaman et al., 2014).

**FIGURE 5:**
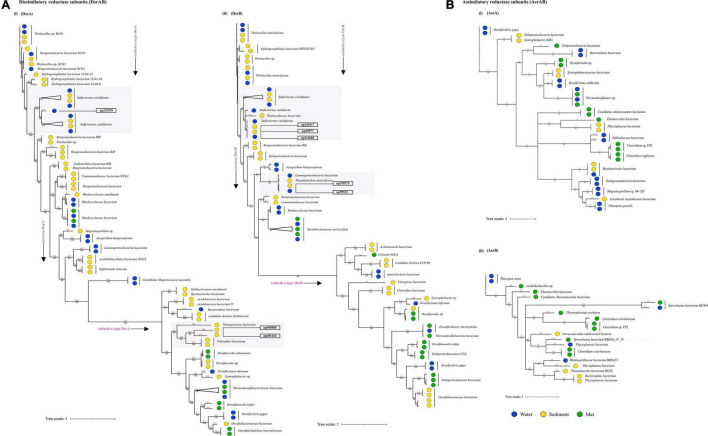
Divergence estimation over time. Reconstruction of the phylogenic tree of optimized full length DsrAB and AsrABC subunits in three habitats using PhyloBayes with the CAT-GTR model. The highlighted squares consist of clades with proteins that were remained unclassified through *nr* database. **(A)** (i) Among, 78 DsrA nodes that showed here the earliest evolution of the rDsr oxidative proteins occurred in *Thiobacillus* sp. (ii) 72 nodes of DsrB proteins with similar results. **(B)** (i) 38 nodes of AsrA and (ii) 21 nodes of AsrB were also compared as a control for branch length shown here.

**FIGURE 6:**
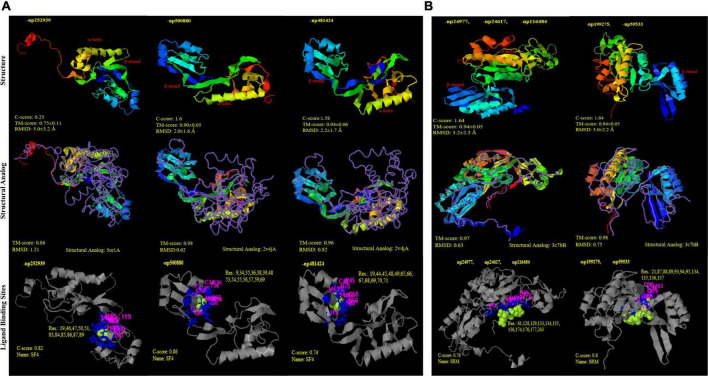
Structural similarity and analogy of unclassified proteins from PDB (Protein data bank) using i-TASSER. **(A)** DsrA protein subunits: (i) np252939 and (ii); (iii) np500880; np481424 showing Tm-align similarity with PDB ids 3or1 and 2v4j (*Desulfovibrio gigas* and *D. vulgaris)*, respectively; with SF4 (iron S cluster) ligand binding sites **(B)** DsrB protein subunits (i) np24977, np24617, np116484 and (ii) np199275, np59533 showing Tm-align similarity with PDB ids 3c7b; with siroheme ligand binding sites.

Through time–scale evolutionary phylogeny of the sequences of DsrAB and AsrAB identified from the metagenomes, we determine the most basal and earliest evolved lineages involved in dsr and *rsdr* pathways (Figure 5 and Supplementary File 7). Our results suggested that both the subunits of the oxidative type reverse-Dsr evolved much earlier than the reductive type Dsr subunits (Figure 5). We used the phylogenetic analysis to further assign taxa to the sequences that showed similarity with yet uncultured bacteria and predicted their structures to gain insights into the more common phylogenetic ancestor of the two Dsr subunits. Interestingly, these sequences of the oxidative type Dsr (rDsr) formed monophyletic clade with a more recently identified genus, *Sulfuritortus* in both DsrA and DsrB phylogenies. Our analysis revealed similar tree topologies with these unassigned sequences forming clade with *Sulfuritortus*, *Thiobacillus*, and *Hydrogenophilales bacterium* in both DsrA (*n* = 1) and DsrB (*n* = 3). Although the sequences were similar to *Thiobacillus* phylogenetically, prediction of their structures revealed DsrA to be highly similar to that of *Desulfovibrio gigas* (TM score = 0.75; analog TM = 0.86; C-score = 0.25, 1.6, and 1.58; Figure 6) and structures of DsrB proteins aligned most closely with *Archaeoglobus fulgidus* (TM score = 0.94; analog TM = 0.97; C-score = 1.64; Supplementary File 9). While the other DsrB subunit (*n* = 2) formed clades with *Thioalkalivibrio* (β-*Proteobacteria*) also showing similarity to *A. fulgidus* (TM score = 0.94; analog TM = 0.98; C-score = 1.64). In the reductive type Dsr clades, two DsrA sequences from uncultured bacteria formed clades with *Nitrospiraceae* and Chloroflexi bacteria (Figure 5). However, a consistent observation was seen in the similarity of all these structures of DsrA proteins with *Desulfovibrio vulgaris* (TM score = 0.94; analog TM = 0.96; C-score = 1.58).

## Discussion

Hot water is continually discharged from a major outlet at Khirganga from where it deposits S upon microbial mats over the sediments along its course (Sharma et al., 2004). Microscopic analysis showed that cyanobacteria are widely distributed in mats and sedimentary deposits of thermal springs (van Gemerden, 1993; Podar et al., 2020). The physio-chemical data signified that the hot spring waters emerges from the confluence of rivers Parvati and Beas have high concentrations of chlorides and sulfates that are characteristic of majority of other hot springs in the Himalayan ranges (Cinti et al., 2009; Sangwan et al., 2015). The alpha diversity was higher in the water samples than microbial mat and sediments as has been reported previously (Ghilamicael et al., 2017; Nagar et al., 2021). Species richness as rarefaction curves obtained for all samples attained a plateau indicating optimum metagenomic sequencing data and sampling of a reasonable number of species for all metagenomes. The Bray–Curtis index calculated and plotted using non-metric multidimensional scaling (NMDS) demonstrated a significant difference in the beta-diversity of all three habitats at phyla level (PERMANOVA; *p* < 0.01). Abundance of class *γ-Proteobacteria* in microbial mats significantly distinguished latter from the other two habitats. Similar results have been reported in previous studies (Selvarajan et al., 2018; Pohlner et al., 2019). Communities in sediment and water samples may be varied from each other majorly due to differences in the abundance of δ-*Proteobacteria*. The dominance of aerobic and facultative anaerobic bacteria like *Pseudomonas* in all three habitats could be possible due to mesothermic environment and natural subsurface water hydrodynamics (Nazina et al., 2005). The other taxa that are often found associated with mat deposits are active biofilm producers that use adherence to the surface as a strategy to survive, evolve, and to cope with various abiotic stresses at such extreme habitats (López et al., 2006; Wright et al., 2013). In contrast, abundance of SOB and SRB in sediment and water was observed (Agostino and Rosenbaum, 2018). These SOB and SRB are usually categorized as lithoautotrophs that play key microbial role in biogeochemical cycling of S in various habitats. In general, hydrogen sulfide account for the S present in the underground geothermal waters originating from pyrites or leaching of other sulfides by deep hypothermal waters (Picard et al., 2016). Sulfide (S^2–^) is oxidized to sulfate (SO_4_^2–^) as the water rises to the surface and under mild oxidizing conditions, sulfide is only oxidized to sulfate or S dioxide (Picard et al., 2016; Wu et al., 2021). The results provided pertinent information on the geochemical composition of the three habitats to be correlated with the microbial diversity and community functions. More importantly, high concentrations of sulfate ions in microbial mats and sedimentary deposits supported the hypothesis of a key role of the bacterial S cycle in sustaining the microbial community at the hot water spring. Sulfur oxidizing bacteria oxidize the reduced S compounds such as hydrogen sulfide (H_2_S), elemental S (S^0^), sulfite (${\text{SO}}_{3}^{2-}$), thiosulfate (S_2_O_3_^2–^), and various polythionates (S_*n*_O_6_^2–^ or -S_*n*_O^6–^) into sulfate (SO_4_^2–^). On the contrary, SO_4_^2–^ can serve as an electron acceptor of SRB under anaerobic conditions, and they reduce the SO_4_^2–^ and other oxidized S compounds (S_2_O_3_^2–^, SO_3_^2–^, and S^0^) into H_2_S (Agostino and Rosenbaum, 2018). The abundance of SOB such as *Thioalkalivibrio* and *Burkholderia* as well as SRB such as *Desulfobulbaceae unclassified* and *Desulfovibrio* in spring is not surprising as high levels of sulfate dominate the site and relative abundance of these bacteria provide evidence of an active S cycling mediated by microbial communities. The enriched diversity for Sox and sulfate reduction as well as the geochemical analysis of sulfide rich habitats compelled us to mine the regulatory genes involved in the different pathways of S cycle. In natural system, the S intermediates are reduced by different bacteria through two different reduction processes, namely, dissimilatory and assimilatory reactions (Vermeij and Kertesz, 1999; Zavarzin, 2008; Figure 3B). In dissimilatory reduction, SRB utilize three enzymes [(ATP sulfurylase (sat), APS reductase (apr), and sulfite reductase (dsr)] to reduce sulfate and produce toxic hydrogen sulfide (Agostino and Rosenbaum, 2018; Kushkevych et al., 2020). On contrary, sulfate is assimilated into organic compounds under assimilatory process (Kushkevych et al., 2020).

Based on sequence similarity, majority of the AsrABC genes (assimilatory) that were taxonomically related to SRB could be distinguished from the rest of the sequences that were not identified as either SOB or SRB. Thus, assimilatory reduction was diverged among the SRB communities while those for dissimilatory pathway were rather conserved at the site. Previous reports of sequence comparisons have confirmed that DsrAB, Dsr, to be highly conserved enzyme that could serve as marker gene for SRBs (Loy et al., 2009). The DsrAB and AprAB enzymes were contributed by both SOB and SRB with syntrophic interaction which suggests for the presence of reverse dsr (rdsr) mediated oxidation of S substrates in addition to dissimilatory reduction (Kumar et al., 2017). The inherent complexity of S-based metabolic network revealed that there are controlled mutation rates in dsrAB genes in presence or increased selective pressure of contamination and extreme conditions. A relatively high diversity of the other sulfate disproportionation proteins in all three segments unveiled the high nutritional demands and efficiency of the microbes toward uptake of a wide range of structurally and chemically diverse amino acid side chains from environment (Talwar et al., 2020). The syntropy of SOB and SRB prevailing in anoxic and anaerobic conditions governs the dissimilatory S metabolism (oxidation and reduction parallelly) and indirectly promoting the growth of diverse microbes in this natural ecosystem (Bhatnagar et al., 2020).

It was noted that mutation rates were low in all subunits of Dsr proteins which we readily analyzed to trace down the ancestor among SOBs or SRBs in dissimilatory and reverse dissimilatory (rdsr) pathways. The result of the time–scale phylogeny suggested that an increase in substitution rate in both subunits of DSR might have occurred on the branch connecting δ-*Proteobacteria* to all other taxa as observed from the branch lengths. The different rates of substitution of the two DSR subunits has this far only been reported in δ-*Proteobacteria* lineage (Wagner et al., 1998). The templates of these *dsrAB* genes have potential to study the genotypic and phenotypic traits in SRPs and the dissimilatory S metabolism processes which will expand the gene-environment interaction mechanism. Also, prior analysis has proved that the evolution of *dsrAB* have been influenced by LGT only among major taxonomic lineages (Klein et al., 2001; Müller et al., 2015) but the findings here provide evidence of independent multiple LGT events distributed throughout the dissimilatory gene clusters. Currently, the time-scale study of this site cannot produce evidence of the progenitor lineages, as the evolutionary history of dissimilatory reduction is complex and yet ascertain. Although it had provided information of the earliest lineage where sulfate/sulfite oxidation and reduction appeared. The genus of SOB currently has few recognized species and is closely related to the members of genus *Thiobacillus* (Kojima et al., 2017). Through structural homology, we predicted that the genus derives its two subunits of Dsr from different ancestors. A plausible explanation for this is observed in the previous reports of high sequence homology between the Dsr of *A. fulgidus* and *D. vulgaris* that suggest a common origin of archaeal and bacterial DSRs or their HGT (Karkhoff-Schweizer et al., 1995). In addition to homology in their sequences, the evolutionary distance separating the enzymes from *A. fulgidus* and *D. vulgaris* was deciphered. For DsrB subunits, the archaeal and bacterial sequences were not particularly distant; such that the branches with structural homology to *A. fulgidus* were approximately the same length as branches leading to bacteria such as *Thiobacillus*, *Thioalkalivibrio*, and β-*Proteobacteria bacterium* (Larsen et al., 1999).

## Conclusion

The mesothermic hot spring have been composed of a diverse group of microbes (Bacteria and Archaea) and genotypes (*dsrAB*) that could be screened out as novel thermozymes that cannot be underestimated. From the results, it could be concluded that the microbial community functions were distinguished in microbial mat from water and sediment. Here, the genomic repertoire suggested the ongoing specific adaptations to cope up with extreme values of sulfide content in this ecological setting. The S metabolic pathways are completed where inorganic S compounds being the main source for SRB releasing toxicity in the form of sulfides (S^2–^). The sulfate reduction profiling in all three habitats reveals dissimilatory sulfate reduction process (*dsrAB*) is active than assimilatory sulfate reduction (*asrAB*). Later, the genes involved in S reduction/oxidation were classified and belong mostly to *Proteobacteria* with maximum homologous proteins classified in anoxygenic SOBs. In all S disproportionation proteins, the sulfite reductase DsrAB proteins showed conserved behavior with 0/1 isolated nodes that have been signified as phylogenetic markers for SRBs. The evolutionary phylogenetic analysis showed that the oxidative rDsr were the earliest than the reductive Dsr which may predict that the condition with more sulfides oxidized in more sulfates, directing SRB to perform dissimilatory reduction later. Phylogenetic clades of DsrAB proteins showed unanimous distribution of taxa except the δ-*Proteobacteria* which could be the reason for occurring LGT to other phyla. On the basis of structural alignments, the lineages with unclassified clades have shown different analogy in both Dsr subunits where DsrB derived from Archaea and DsrA are δ-*Proteobacteria* in origin at this mesothermic niche. The stabilization and evolutionary time–scale phylogeny of DsrAB revealing a positive syntrophic relationship between SOB and SRB. These thermophilic microbial inhabitants are very crucial in expanding the metal toxification, ion exchange, and biogeochemical cycling of the elements.

## Data Availability Statement

The datasets presented in this study can be found in online repositories. The names of the repository/repositories and accession number(s) can be found in the article/Supplementary Material.

## Author Contributions

SN: conceptualization, methodology, investigation, formal analysis, data curation, methodology, writing—original draft, review, and editing. CT: formal analysis, data curation, methodology, writing—original draft, review, and editing. MM-H and H-HR: formal analysis, writing—review, and editing. MS: writing—review and editing. RL and RN: conceptualization, Writing—review, and editing. All authors contributed to the article and approved the submitted version.

## Conflict of Interest

The authors declare that the research was conducted in the absence of any commercial or financial relationships that could be construed as a potential conflict of interest.

## Publisher’s Note

All claims expressed in this article are solely those of the authors and do not necessarily represent those of their affiliated organizations, or those of the publisher, the editors and the reviewers. Any product that may be evaluated in this article, or claim that may be made by its manufacturer, is not guaranteed or endorsed by the publisher.

## Supplementary Material

Supplementary File 1 NCBI accession and *in-situ* measures of temperatures, pH, and elemental analysis of each sample from three studied niches.

Supplementary File 2 **(A)** Rarefaction curves based on measures of alpha diversity species richness calculated for each of the six samples. **(B)** The interquartile range of alpha diversity measures based on Shannon indices. **(C)** Non-metric multidimensional scaling plots based on Bray–Curtis indices of community dissimilarities in the six samples types (*p* < 0.05, PERMANOVA).

Supplementary File 3 The list of all metabolic gene families in each sample (KgM1, KgM2, KgS1, KgS2, KgW1, and KgW2) and the RPKs abundances of taxa (*Proteobacteria, Bacteroidetes*, etc.) summarized in details.

Supplementary File 4 Detailed information of percentage reconstruction of metabolic pathways; Common and unique pathways in all six samples; Identification sources for 19 important proteins involved in S disproportionation using TIGRfam and Pfam conserved domain database, *x* indicates no hits found. Also, the copy number of 25 S disproportionation proteins recorded.

Supplementary File 5 Details of taxonomic information of 2,413 nodes of 19 S cycle genes identified from nr NCBI microbial proteins database.

Supplementary File 6 Habitat vs. habitat *dN*/*dS* values of the S cycling genes.

Supplementary File 7 Sequence alignment of DsrA, DsrB, AsrA, and AsrB proteins identified in this study.

Supplementary File 8 Phylogenetic analysis of DsrAB proteins: showing percentage similarity with taxa on NCBI *nr* database. Few newly identified lineages were marked in red. In DsrAB, maximum number of LGT events were occurred in Proteobacteria class itself *clade* **(A2,B2)** while the independent events of LGT toward different phyla were occurred through δ-Proteobacteria depicted in *clade* **(A1,B1)**.

Supplementary File 9 Structural models for other ligand binding sites of unclassified proteins of DsrA and DsrB subunits.
