# Supplementary figures and images for: Microbial Ecology of Sulfur Biogeochemical Cycling at a Mesothermal Hot Spring Atop Northern Himalayas, India
Source: Front Microbiol. 2022 Apr 13;13:848010. doi: 10.3389/fmicb.2022.848010 (PMC9044081; doi:10.3389/fmicb.2022.848010)

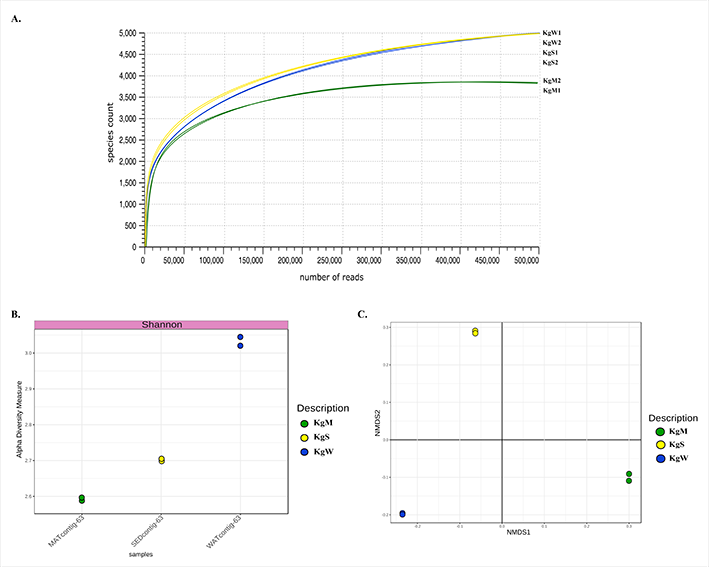

Supplement: Supplementary File 2 — (A) Rarefaction curves based on measures of alpha diversity species richness calculated for each of the six samples. (B) The interquartile range of alpha diversity measures based on Shannon indices. (C) Non-metric multidimensional scaling plots based on Bray–Curtis indices of community dissimilarities in the six samples types (p < 0.05, PERMANOVA). [file Image_1.TIF]

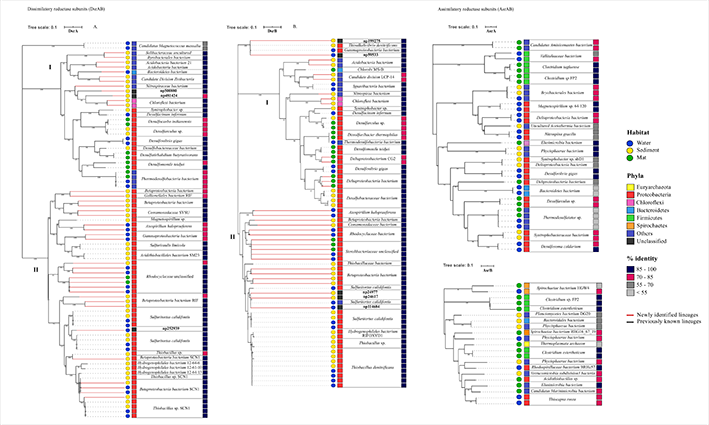

Supplement: Supplementary file 6 [file Image_2.TIF]

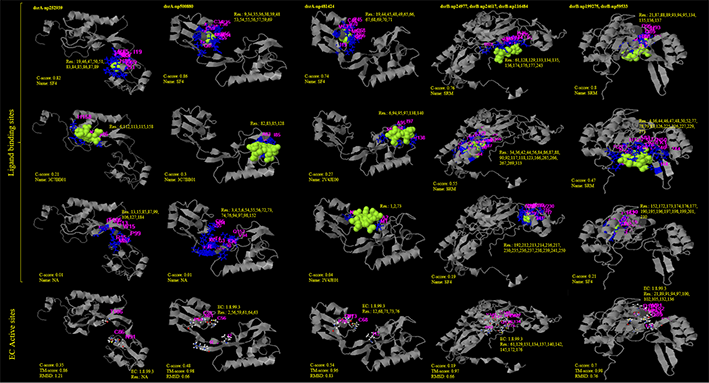

Supplement: Supplementary file 7 [file Image_3.TIF]
